# ERP Evidence for Cross-Modal Effects on Attractiveness Perception

**DOI:** 10.3390/brainsci16040402

**Published:** 2026-04-09

**Authors:** Qi Zhang, Linyan Wang, Weijun Li

**Affiliations:** 1 Faculty of Education, Beijing Normal University, Beijing 100875, China; littlesun1995@163.com; 2 Institute of Psychological and Brain Sciences, Liaoning Normal University, Dalian 116029, China; wlyan5588@163.com

**Keywords:** facial attractiveness, vocal attractiveness, audiovisual integration, cross-modal effect, ERPs

## Abstract

**Highlights:**

**What are the main findings?**

• Audiovisual presentation of faces and voices facilitated attractiveness judgments compared with unimodal voice presentation.
• ERP results revealed that cross-modal attractiveness congruency modulated neural processing at both early perceptual (N1) and later evaluative (P3) stages.

**What are the implications of the main findings?**

• The findings demonstrate how facial information influences the neural processing of vocal attractiveness during multisensory social perception.
• This study provides electrophysiological evidence for the temporal dynamics of audiovisual integration in attractiveness evaluation.

**Abstract:**

Background: Attractiveness plays an important role in social interactions. However, it remains unclear whether presenting attractiveness information across multiple sensory modalities facilitates attractiveness evaluation, and how cross-modal congruency modulates this process. Methods: The present study used event-related potentials (ERPs) to investigate these questions. Participants judged the attractiveness of voices presented alone or paired with faces that were congruent or incongruent in attractiveness. Results: Significant differences were found between unimodal and audiovisual conditions, as well as between congruent and incongruent pairs, during both early perceptual (N1) and later evaluative (P3) stages. Both audiovisual conditions elicited larger N1 amplitudes than the auditory-only condition, and congruent pairs produced larger N1 amplitudes than incongruent pairs. At a later stage, the auditory-only condition produced larger P3 amplitudes than the audiovisual conditions. Furthermore, the interaction between voice attractiveness and visual context on P3 amplitudes was significant. Audiovisual incongruent pairs elicited larger P3 amplitudes than congruent pairs, but only when the voice was unattractive. Conclusions: The results demonstrate that redundant visual cues of attractiveness both accelerate and alter the perception of auditory attractiveness. These audiovisual integration effects occur across different processing stages and may reflect enhanced processing efficiency in multisensory social perception.

## 1. Introduction

Attractiveness plays a central role in social interaction, shaping emotional responses and guiding approach or avoidance tendencies [1]. People rapidly extract attractiveness cues from others through one or more sensory channels, with vision and audition (such as faces and voices) being the two primary channels through which attractiveness information is conveyed. These cues influence judgments of mate value [2,3], personality and social competence [4,5], and even moral character [6]. As a result, attractiveness has meaningful consequences in mate choice [7,8], employment decisions [9,10] and economic behavior [11,12]. However, access to attractiveness cues is often constrained in daily life. In contexts such as phone interviews, noisy social settings, or limited attentional states, individuals may be forced to rely on only one modality. Interestingly, prior research has shown that attractiveness conveyed through a single modality (e.g., face or voice) may exert stronger effects on impression formation than when face and voice are presented together [13,14]. These findings raise two key questions: whether multimodal input enhances attractiveness perception relative to unimodal input, and whether cross-modal congruency in attractiveness modulates this processing.

Multisensory integration refers to the process by which the brain combines information from different sensory modalities into a unified and coherent percept [15,16]. This process is characterized as an automatic process and often yields the redundant-target effect, whereby responses to redundant bimodal stimuli are significantly faster than those to unimodal stimuli [17,18,19]. Existing studies indicate close interactions between the auditory and visual systems: auditory signals can enhance visual perception [20,21,22], and visual information can likewise modulate auditory recognition [23,24]. In speech perception, when auditory speech is presented synchronously with corresponding lip movements, speech recognition accuracy is significantly improved [25,26,27]. Such cross-modal binding facilitates perception when cues are congruent, but may induce conflict or perceptual distortion when they are incongruent, as illustrated by sound-induced visual illusions and the McGurk effect [28,29]. In general, cross-modal information is more likely to be perceived as originating from the same event and to be integrated when it is consistent in space, time, or semantic meaning [2,30,31,32].

Faces and voices provide visual and auditory cues of attractiveness, respectively. Facial averageness, symmetry, and sexual dimorphism are important features influencing facial attractiveness [8,33], while vocal attractiveness can be effectively predicted by acoustic properties such as fundamental frequency, formant frequency, and harmonic-to-noise ratio [34,35,36,37]. Behavioral studies have shown that when face and voice attractiveness are presented simultaneously, they interact during integration and jointly shape subjective evaluations of overall attractiveness [38,39]. When the two modalities are incongruent in attractiveness, the evaluation tends to be biased toward the visual information; that is, the “attractive face + unattractive voice” combination is rated more attractive than the opposite pairing [39], indicating a dominance of visual cues in attractiveness processing. Even when the task requires judging only voice attractiveness, facial cues are automatically activated and influence the evaluation, whereas the reverse influence is weaker [40,41]. Although some studies report no significant interaction, the impact of facial attractiveness is estimated to be approximately 4 times stronger than that of vocal attractiveness [38,42].

ERPs offer high temporal precision and are therefore well suited for uncovering the implicit processing dynamics underlying attractiveness perception. Previous studies have examined neural responses to vocal attractiveness alone as well as to the integration of facial and vocal attractiveness [43,44]. Zhang et al. [43] showed that attractive voices elicited enhanced N1 responses across both implicit and explicit tasks, whereas a late LPC enhancement emerged only when attractiveness was explicitly evaluated, suggesting that early vocal attractiveness processing is automatic, while later evaluative stages rely on attentional control. Using a Stroop-like audiovisual paradigm, Liu et al. [44] reported that facial attractiveness exerted a robust influence on voice-attractiveness judgments during both early and late stages, whereas the reverse influence was restricted to early processing, demonstrating asymmetric cross-modal effects and a perceptual advantage for visual cues. However, these studies employed either unimodal or bimodal designs, and thus cannot reveal the differences in attractiveness processing between unimodal and multimodal conditions. Consequently, it remains unknown whether redundant information from an additional modality modulates perceived attractiveness in the primary modality.

In ERP research, the effects of audiovisual integration are often indexed by the early auditory N1 component. The fronto-central N1 is associated with early encoding of auditory features and the detection of motivationally salient input [45]. Studies of audiovisual speech and emotional processing consistently report reduced N1 amplitudes for audiovisual stimuli relative to auditory-only stimuli [46,47,48,49,50,51,52], and this N1 suppression is often regarded as evidence of facilitated audiovisual integration. Alternatively, the predictive coding hypothesis proposes that N1 suppression arises because visual cues enhance the predictability of auditory events [53,54,55], reflecting reduced uncertainty and lowered computational demands in auditory cortical regions [56]. Furthermore, audiovisual congruency modulates N1 amplitudes. When visual cues are congruent with the auditory input (e.g., in emotional meaning), N1 amplitudes are significantly larger than in incongruent conditions, indicating enhanced early attentional allocation and sensory feature extraction [57,58,59]. However, other studies have reported no significant congruency effects [52,53], suggesting that this effect is not stable and may be moderated by stimulus type and attribute characteristics (e.g., emotion versus speech, affective valence, type of visual signal). Thus, existing findings indicate that N1 may reflect either integration-related facilitation or cross-modal prediction processes, but whether attractiveness as a social cue induces similar N1 effects in audiovisual integration remains unresolved.

The P3 component reflects later-stage evaluative processing, including stimulus categorization and the allocation of controlled attentional resources [60]. Evidence from speech, emotional, and music-based audiovisual paradigms shows that P3 is sensitive to cross-modal congruency [59,61,62], supporting its role in the re-evaluation of matched versus conflicting multisensory cues. Thus, P3 provides a suitable index for examining later integration processes in attractiveness perception.

Taken together, the present study compared unimodal (voices only) and bimodal (voices and faces) attractiveness processing and examined the role of audiovisual congruency using behavioral measures and ERPs. Based on the robust redundancy effect observed in multisensory integration, we predicted more efficient processing in bimodal conditions, reflected in faster behavioral judgments and early N1 modulation. Additionally, given the weak correspondence between facial and vocal attractiveness [42,63], audiovisual congruency may exert distinct effects on the integration process. Therefore, we will explore this through an exploratory analysis comparing N1 and P3 amplitudes.

## 2. Method

### 2.1. Participants

A power prior analysis was conducted using G*Power 3.1, which showed that, with a significance level of *α* = 0.05 and a medium effect size (*f* = 0.25), a minimum of 28 participants would be required to achieve 0.80 statistical power. Considering that the experimental stimuli included both male and female faces and voices, we recruited an equal number of male and female participants. A total of 34 heterosexual undergraduate students were recruited from Liaoning Normal University. Due to excessive EEG artifacts, data from one male and two female participants were excluded. The final sample consisted of 31 participants (15 females,16 males, *M*_age_ = 21.26 years, *SD* = 2.06). All participants were native Mandarin speakers with normal or corrected-to-normal vision, normal hearing, and no history of neurological or psychiatric disorders. Written informed consent was obtained from all participants. The study was approved by the Research Ethics Committee of Liaoning Normal University and conducted in accordance with the Declaration of Helsinki. Participants were reimbursed for their participation.

### 2.2. Stimuli

The facial stimuli were selected from the facial stimulus set developed by Yang et al. [64], which comprised 30 highly attractive and 30 less attractive faces, with an equal number of male and female models. All images depicted neutral expressions in a frontal view with direct gaze, and external features (e.g., hair contours and ears) were digitally removed. Each image was cropped to 260 × 300 pixels and presented in grayscale.

The vocal stimuli were recorded from native Mandarin speakers and edited using a professional digital audio workstation (Adobe Audition CS6, version 5.0), with sampling parameters set to 44,100 Hz and 16-bit resolution. All audio files were saved in .wav format. After excluding recordings with noise or noticeable accents, 65 male and 66 female tokens of the Mandarin greeting “nihao” (“hello”) were retained. Using Praat software (http://www.fon.hum.uva.nl/praat/, accessed on 1 October 2017), all voice segments were standardized to 600 ms in duration and normalized to an intensity of 70 dB SPL.

To ensure the validity and reliability of the attractiveness manipulation, an independent group of 41 university students (21 males, 20 females) was recruited to rate the attractiveness of all faces and voices using a 7-point Likert scale. Based on the rating results, stimuli were categorized according to gender (female, male) and attractiveness level (attractive, unattractive). For the final experimental set, 12 stimuli were retained for each condition within each modality: female attractive faces (*M* ± *SD* = 5.11 ± 0.42), female unattractive faces (3.08 ± 0.37), male attractive faces (4.83 ± 0.34), male unattractive faces (2.60 ± 0.67), female attractive voices (4.87 ± 0.29), female unattractive voices (3.27 ± 0.23), male attractive voices (5.15 ± 0.25), and male unattractive voices (3.24 ± 0.22). The mean attractiveness ratings significantly differed between the attractive and unattractive categories within each gender group (all *p*s < 0.001). These results serve as the normative validation of attractiveness manipulation for the materials used in this study.

### 2.3. Procedure

The experiment included a unimodal auditory condition and two bimodal audiovisual conditions. In the unimodal condition, participants were presented with voice stimuli only. In the bimodal conditions, a face and a voice were presented simultaneously, with the gender of the face always matching that of the voice. The two modalities could convey either congruent or incongruent levels of attractiveness. Accordingly, the final design comprised 6 experimental conditions: (1) attractive voices only, (2) unattractive voices only, (3) attractive voices paired with attractive faces (congruent), (4) unattractive voices paired with unattractive faces (congruent), (5) attractive voices paired with unattractive faces (incongruent), and (6) unattractive voices paired with attractive faces (incongruent).

**Figure 1:**
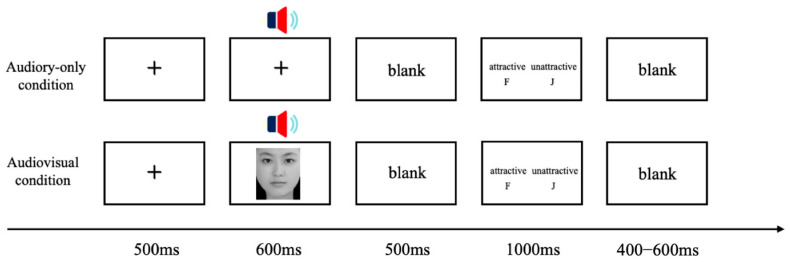
The flowchart of a single trial in the experiment.

The experiment was conducted in a quiet, dimly lit, and electromagnetically shielded room. Participants were seated approximately 90 cm from the display monitor. Visual stimuli were presented at the center of the screen, and auditory stimuli were delivered binaurally through headphones at a volume adjusted to a comfortable listening level. All stimuli were presented in a pseudorandom order, ensuring that no more than two stimuli of the same type appeared consecutively. Each trial followed the sequence illustrated in Figure 1. A fixation cross (“+”) was first presented for 300 ms, followed by the stimulus presentation. In the auditory-only condition, only the voice stimulus was played, whereas in the audiovisual condition, the face and voice were presented simultaneously. The duration of each stimulus matched the duration of its corresponding voice clip. After stimulus offset, a blank screen was shown for 500 ms. Participants were instructed to judge the attractiveness of the voice and respond within 1000 ms using the keyboard, pressing “F” for “attractive” and “J” for “unattractive.” The response-key mapping was counterbalanced across participants. An inter-trial interval of 300–500 ms of a blank screen was inserted between trials. Participants were instructed to maintain fixation on the center of the screen (either the fixation cross or the face) and to respond as quickly and accurately as possible. Prior to the formal experiment, they completed several practice trials to familiarize themselves with the task. Each participant was presented with stimuli from both genders. Each condition contained 96 trials, resulting in a total of 576 trials. The trials were divided into six blocks (two auditory blocks and four audiovisual blocks), with block order counterbalanced across participants. Each block lasted approximately six minutes, and participants could take breaks between blocks. The entire experiment lasted approximately 40 min.

### 2.4. EEG Recording and Processing

EEG data were recorded using a 64-channel electrode cap arranged according to the international 10–20 system (Brain Products GmbH, Gilching, Germany). Signals were sampled at 500 Hz and referenced online to FCz. During recording, a band-pass filter of 0.01–100 Hz was applied, and electrode impedance was maintained below 5 kΩ. Offline, data were re-referenced to the average of the left and right mastoids. EEG preprocessing was conducted in MATLAB R2022a using EEGLAB v2023.0. The data were band-pass filtered between 0.1 and 30 Hz, and obvious artifacts (e.g., muscle and movement artifacts) were manually removed. Independent Component Analysis was applied to identify and correct ocular and other residual artifacts [65]. Epochs exceeding ±80 μV were rejected. Data were segmented into epochs from 200 ms before stimulus onset to 800 ms after onset, and trials with incorrect behavioral responses were excluded. Baseline correction was performed using the 200 ms pre-stimulus interval. After artifact correction and trial exclusion, approximately 81.26% of the total trials were retained for further analysis.

### 2.5. Statistical Analysis

Data analyses were conducted using IBM SPSS Statistics 27. For the behavioral data, following the approach of Liu et al. [44], we analyzed reaction times (RTs) and percent agreement with normative ratings (i.e., the proportion of trials in which participants made choices that were consistent with normative attractiveness ratings). Both RTs and agreement rates were subjected to a 2 (Voice Attractiveness: attractive, unattractive) × 3 (Visual Context: auditory-only, audiovisual congruent, audiovisual incongruent) repeated-measures ANOVA.

For the ERP data, based on visual inspection of the grand average waveforms and prior literature, we analyzed N1 and P3 components. The N1 component (80–120 ms) was quantified as the mean amplitude across fronto-central electrodes (FC1, FCz, FC2, C1, Cz, C2). The P3 component (230–310 ms) was measured at fronto-central electrodes (F1, Fz, F2, FC1, FCz, FC2), consistent with prior studies reporting P3 effects over anterior and central regions [43,66,67]. For each component, mean amplitudes were computed for each participant and experimental condition by averaging values within the corresponding time window and region of interest (ROI). These mean amplitudes were then analyzed using a 2 (Voice Attractiveness: attractive, unattractive) × 3 (Visual Context: auditory-only, audiovisual congruent, audiovisual incongruent) repeated-measures ANOVA, identical to that used for the behavioral data. Greenhouse–Geisser corrections were applied when the sphericity assumption was violated, and only corrected *p*-values are reported. Significant main effects and interactions were further examined using post hoc pairwise comparisons with Bonferroni adjustment for multiple comparisons.

## 3. Results

### 3.1. Performance

**Figure 2:**
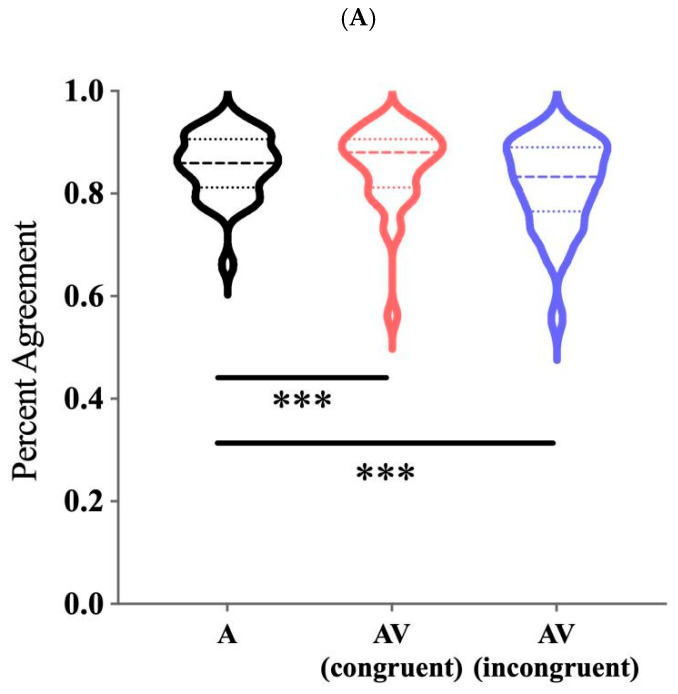

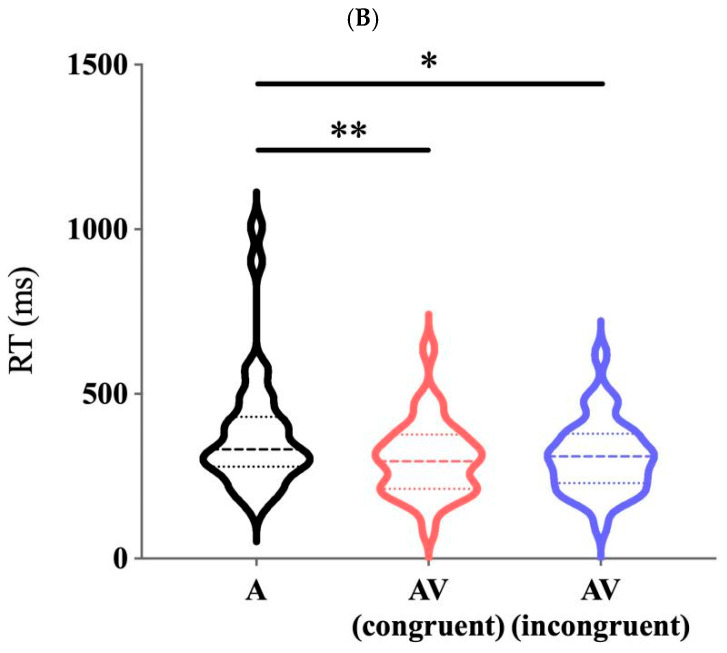
Percent agreement with normative ratings (**A**) and RTs (**B**) in the three visual context conditions. * *p* < 0.05; ** *p* < 0.01; *** *p* < 0.001. A: auditory-only; AV: audiovisual.

For percent agreement, the main effect of visual context was significant, *F*(2, 60) = 15.303, *p* < 0.001, *η_p_*^2^ = 0.338. The agreement rate was significantly higher in both the auditory-only condition (0.855 ± 0.011, *p* < 0.001) and the audiovisual congruent condition (0.855 ± 0.014, *p* < 0.001) compared to the audiovisual incongruent condition (0.818 ± 0.016) (see Figure 2). The main effect of voice attractiveness and the interaction were not significant.

For RTs, the main effect of visual context was significant, *F*(2, 60) = 5.525, *p* = 0.009, *η_p_*^2^ = 0.276. Reaction times were significantly shorter in the audiovisual congruent (302.146 ± 20.211 ms, *p* = 0.007) and audiovisual incongruent conditions (308.578 ± 19.835 ms, *p* = 0.013) than in the auditory-only condition (382.913 ± 33.452 ms). The difference between the two audiovisual conditions was not significant (*p* = 0.552) (see Figure 2). The main effect of voice attractiveness and the interaction were not significant.

### 3.2. ERP Results

#### 3.2.1. N1 (80–120 ms)

**Figure 3:**
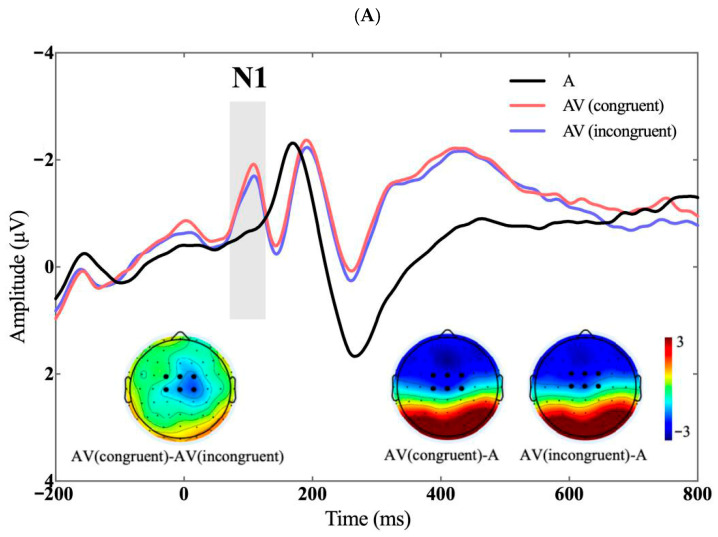

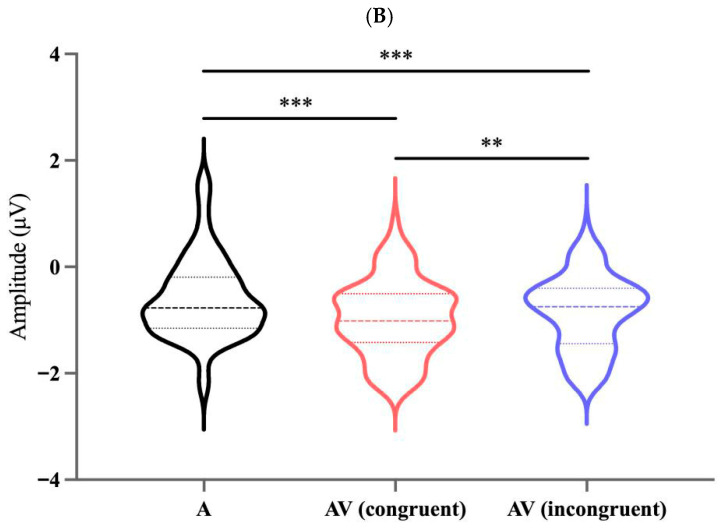
N1 (80–120 ms). (**A**) The top panel shows ERP waveshapes averaged across electrodes in the N1 ROI; inserts show the difference topographies during the time window of the N1. (**B**) The bottom panel shows violin plots of N1 amplitudes in the three visual context conditions. ** *p* < 0.01; *** *p* < 0.001. A: auditory-only; AV: audiovisual.

A significant main effect of visual context was observed on N1 amplitudes, *F*(2, 60) = 25.095, *p* < 0.001, *η_p_*^2^ = 0.455. The audiovisual congruent condition elicited larger N1 amplitudes than the auditory-only condition (−1.597 ± 0.239 versus −0.649 ± 0.135 μV), *p* < 0.001. Similarly, the audiovisual incongruent condition elicited larger N1 amplitudes than the auditory-only condition (−1.428 ± 0.237 versus −0.649 ± 0.135 μV), *p* < 0.001. Moreover, N1 amplitudes in the audiovisual congruent condition were significantly larger than those in the audiovisual incongruent condition (−1.597 ± 0.239 versus −1.428 ± 0.237 μV), *p* = 0.021, see Figure 3. The main effect of voice attractiveness and the interaction were not significant.

#### 3.2.2. P3 (230–310 ms)

**Figure 4:**
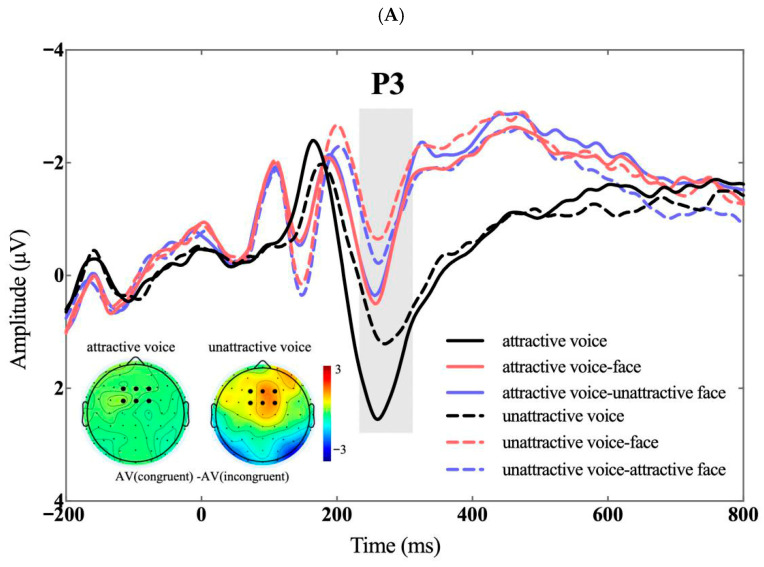

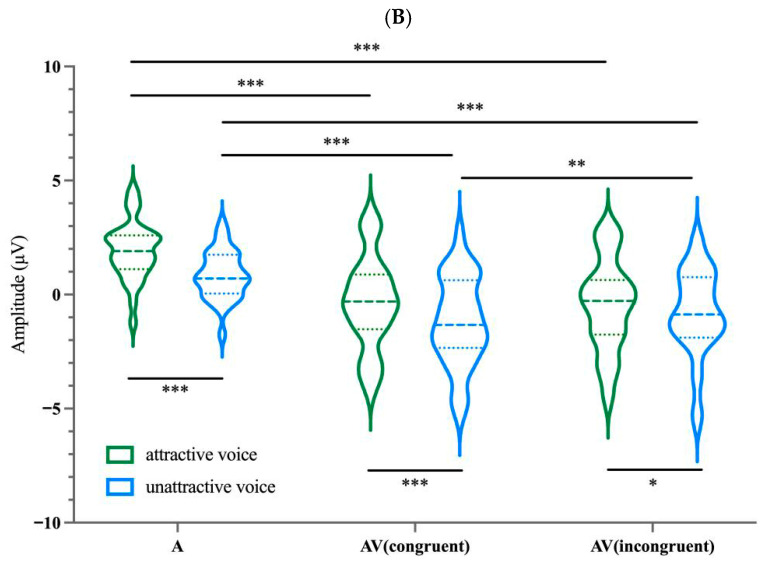
P3 (230–310 ms). (**A**) The top panel shows ERP waveshapes averaged across electrodes in the P3 ROI; inserts show the difference topographies during the time window of the P3. (**B**) The bottom panel shows violin plots of P3 amplitudes in all conditions. * *p* < 0.05; ** *p* < 0.01; *** *p* < 0.001. A: auditory-only; AV: audiovisual.

There was a significant main effect of voice attractiveness on P3 amplitudes, *F*(1, 30) = 57.918, *p* < 0.001, *η_p_*^2^ = 0.659, with attractive voices eliciting larger P3 amplitudes than unattractive voices (0.375 ± 0.291 versus −0.384 ± 0.263 μV). A significant main effect of visual context was also found, *F*(2, 60) = 50.820, *p* < 0.001, *η_p_*^2^ = 0.629. The auditory-only condition elicited significantly larger P3 amplitudes than both the audiovisual congruent (1.393 ± 0.205 versus −0.774 ± 0.350 μV), *p* < 0.001, and audiovisual incongruent conditions (1.393 ± 0.205 versus −0.633 ± 0.342 μV), *p* < 0.001. The difference between the two audiovisual conditions was not significant, *p* = 0.319, see Figure 4.

In addition, the interaction between voice attractiveness and visual context was significant, *F*(2, 60) = 9.626, *p* < 0.001, *η_p_*^2^ = 0.243. Simple effect analyses showed that, for attractive voices, the auditory-only condition elicited larger P3 amplitudes than both audiovisual congruent and audiovisual incongruent conditions (1.935 ± 0.229 versus −0.356 ± 0.364 μV, *p* < 0.001; 1.935 ± 0.229 versus −0.455 ± 0.360 μV, *p* < 0.001), with no significant difference between the two audiovisual conditions (*p* = 1.000). For unattractive voices, the auditory-only condition again elicited larger P3 amplitudes than the audiovisual congruent and audiovisual incongruent conditions (0.851 ± 0.190 versus −1.192 ± 0.352 μV, *p* < 0.001; 0.851 ± 0.190 versus −0.812 ± 0.343 μV, *p* < 0.001). Moreover, the audiovisual incongruent condition elicited significantly larger P3 amplitudes than the audiovisual congruent condition (−0.812 ± 0.343 versus −1.192 ± 0.352 μV), *p* = 0.008, see Figure 4.

## 4. Discussion

Using ERPs, the present study directly compared unimodal and multimodal attractiveness processing and examined how audiovisual congruency shapes the time course of voice attractiveness perception. The findings demonstrate that both redundant visual information and the degree of congruency between facial and vocal attractiveness modulate voice attractiveness processing, with significant effects emerging at both early perceptual and later evaluative stages. In addition, the influence of audiovisual congruency on the late stage of voice attractiveness processing was moderated by the voice attractiveness level.

### 4.1. Audiovisual Facilitation in Attractiveness Judgments

Behavioral results confirmed the presence of a redundant-target effect in attractiveness perception. In both audiovisual conditions, participants judged voice attractiveness significantly faster than in the auditory-only condition, while maintaining over 80% agreement with normative attractiveness ratings. Notably, in the audiovisual congruent condition, participants responded faster without changing the outcome of attractiveness judgments (compared to the auditory-only condition), suggesting a cross-modal congruency enhancement effect. In contrast, in the audiovisual incongruent condition, although the responses were also faster, the judgments of voice attractiveness showed a significant shift—attractive voices paired with unattractive faces were rated as less attractive, and unattractive voices paired with attractive faces were rated as more attractive. This indicates that visual cues not only enhanced processing efficiency but also biased attractiveness evaluation when facial and vocal signals conflicted.

This pattern aligns with the unity assumption, which posits that observers tend to infer whether cues from different modalities originate from a single source and integrate them when this is presumed [68]. In this study, the visual and auditory stimuli were presented synchronously in time and space, and the face and voice matched in gender, satisfying the temporal, spatial, and semantic conditions for cross-modal integration [69]. This likely led participants to treat the face and voice as originating from the same source, thereby promoting rapid integration of attractiveness cues and improving processing efficiency. Even when attractiveness levels were cross-modal incongruent, participants likely relied on the prior assumption that the stimuli originated from the same individual, resulting in systematic shifts in voice attractiveness perception toward the level of facial attractiveness.

### 4.2. Early Attention and Congruency Effects in N1

A key finding of this study is that audiovisual stimuli elicited significantly larger N1 amplitudes than auditory-only stimuli, indicating that facial information was automatically processed at an early stage, even when it was irrelevant to the task. This supports the view that face processing is mandatory and automatic. However, unlike the commonly observed N1 suppression effect in previous speech or emotional audiovisual integration studies [48,50,51,70,71], the present study observed an enhancement of N1. Our results indicate that audiovisual integration of attractiveness does not conform to the predictive coding hypothesis, which may be due to the fact that cross-modal “conflict” in attractiveness perception does not strongly require resolution. In social interaction, auditory and visual cues for speech or emotion are typically highly correlated (e.g., tone of voice and facial expression conveying the same emotional content). Therefore, inconsistent cross-modal signals tend to induce cognitive conflict (e.g., the McGurk effect), which requires additional cognitive resources to maintain efficient processing and accurate interpretation of the linguistic or emotional meaning. Thus, predictive mechanisms are necessary in speech and emotional processing. In contrast, attractiveness cues primarily influence approach–avoidance tendencies rather than the interpretation of social meaning. Therefore, cross-modal mismatches in attractiveness may not strongly recruit predictive or conflict-monitoring processes. In other words, the motivation reflected by N1 suppression, namely conserving cognitive resources to resolve important cross-modal conflicts, may not exist in attractiveness processing.

The observation of larger N1 amplitudes in the audiovisual compared to the auditory-only condition can be understood in terms of attentional modulation of early sensory processing. It is well established that enhanced attention to auditory stimuli increases auditory cortical activity and leads to enlarged N1 amplitudes within the corresponding time window [72,73]. Consistent with this view, cross-modal attention has also been shown to amplify N1 amplitudes, reflecting facilitated early perceptual processing [74,75,76,77,78]. In the present study, the face presented synchronously with the voice may have acted as a salient visual cue that biased attention toward the auditory input [55]. Supporting this interpretation, fMRI studies have demonstrated that visual attention can spread to simultaneously presented, task-irrelevant auditory stimuli, leading to co-activation of auditory and visual cortices [79]. Accordingly, the audiovisual condition likely involved not only task-driven auditory attention, but also an additional layer of cross-modal attentional allocation induced by the visual stimulus (i.e., faces), resulting in enhanced N1 amplitudes.

Moreover, the finding that attractiveness-congruent audiovisual stimuli elicited larger N1 amplitudes than incongruent stimuli indicates that attractiveness-related information was rapidly extracted from both modalities and preliminarily integrated at an early stage. This N1 congruency effect is consistent with findings in emotional audiovisual integration [51,57,58,59], suggesting that N1 is highly sensitive to the affective content of audiovisual inputs. The present study extends this conclusion to the domain of attractiveness. Taken together, the N1 findings suggest that visual input can automatically enhance attentional allocation to the auditory modality, thereby facilitating early processing of vocal attractiveness. Furthermore, attractiveness-congruent audiovisual stimuli evoke stronger early integration, indicating that attractiveness information can be rapidly matched and integrated across sensory modalities.

### 4.3. Evaluative and Control Processes Reflected in P3

During the P3 time window, attractive voices elicited larger P3 amplitudes than unattractive voices across both unimodal and audiovisual conditions, replicating previous findings [43,80,81]. This confirms that P3 serves as a robust neural index of vocal attractiveness and that this effect remains stable even when visual input is added (provided that attention is directed to the auditory channel). Given that P3 reflects the evaluative and motivational significance of stimuli [82], the enhanced P3 for attractive voices likely arises from their greater sociobiological significance, such as the higher mating value and more positive personality traits associated with individuals with more attractive voices [13,83,84]. Such positive significance may lead to greater motivated attention and cognitive resource allocation.

In addition, we observed a significant main effect of visual context, such that the auditory-only condition elicited larger P3 amplitudes than both audiovisual conditions. This indicates that redundant facial attractiveness cues reduced the cognitive load during the vocal attractiveness evaluation phase. This effect may reflect an adaptive advantage of multisensory integration at later processing stages. Specifically, the presence of a face may enhance attentional allocation and early processing of acoustic features related to vocal attractiveness, allowing the voice to enter the evaluative stage with a clearer and more stable perceptual representation and thus reducing cognitive resource demands. Meanwhile, under bimodal conditions, the brain tends to assume that the face and voice originate from the same source and integrates them by weighting their attractiveness cues according to their reliability or variability, forming a coherent and robust multisensory percept—a process considered an “optimal” integration strategy [85,86,87]. Our findings suggest that this strategy may enhance the efficiency of attractiveness judgment even when facial attractiveness is task-irrelevant, resulting in reduced cognitive resource consumption during later evaluation, reflected in reduced P3 amplitude. Overall, the multisensory effects on P3 highlight the functional significance of multisensory integration in human perception, particularly in facilitating efficient processing of socially relevant information [88,89].

A noteworthy finding is that audiovisual incongruence elicited a larger P3 amplitude than audiovisual congruence only in the low-attractiveness voice condition. This indicates that when the voice is relatively unattractive, the presence of an attractive face incurs additional cognitive effort to resolve cross-modal perceptual conflict. Two mechanisms may account for this effect. First, even when faces are task-irrelevant, they still dominate during attractiveness processing [38,42,44,90]. Especially attractive faces with high sociobiological signaling value are more likely to trigger automatic processing when cognitive resources are limited [91]. Therefore, the increased P3 amplitude partially reflects the allocation of motivational attention resources driven by attractive faces. Second, incongruent pairings require individuals to suppress their positive bias toward attractive faces to preserve an independent evaluation of vocal attractiveness, engaging additional top-down control, which increases P3 amplitude. Together, these results suggest that late-stage bimodal attractiveness processing reflects a joint contribution of stimulus-driven attention and goal-directed cognitive control [92].

### 4.4. Limitations and Future Directions

Several limitations in our study should be noted. First, we used static facial images paired with dynamic vocal stimuli, which may introduce audiovisual temporal mismatch and affect integration. Although this design allowed tighter experimental control, it may constrain the generalizability of the findings to more naturalistic social interactions. Future studies should employ dynamic, temporally synchronized, and more naturalistic stimuli. Second, only a vocal attractiveness judgment task was used to equate attentional demands across conditions. However, attention in real-world contexts is likely to shift across modalities. Given the complex interaction between attention and audiovisual integration [93], future research should incorporate visual-only conditions, independent facial judgments, and holistic evaluations to better characterize attentional modulation and its temporal dynamics. Third, the sample size was not optimized for between-group comparisons; thus, factors such as gender were not included in the main analyses. Larger and more balanced samples are needed to examine individual differences in audiovisual integration of attractiveness. Finally, the present analyses were limited to scalp-level ERP effects. Future work could combine source-level and connectivity-based approaches to characterize both the cortical generators and the network dynamics underlying multimodal attractiveness processing.

## 5. Conclusions

In conclusion, the present study used ERPs to reveal neural differences between unimodal and bimodal attractiveness processing. The results showed that at early stages, task-irrelevant facial information was automatically integrated with vocal signals and enhanced perceptual encoding (enhanced N1), with clear audiovisual congruency effects. At later stages, bimodal integration reduced cognitive effort relative to unimodal processing (reduced P3), and the congruency effect emerged only when vocal attractiveness was low. These findings provide new neural evidence for audiovisual integration of human attractiveness and suggest that the presence and level of visual attractiveness cues may influence the objectivity and accuracy of vocal attractiveness judgments.

## Data Availability

The data presented in this study are available on request from the corresponding author due to participant privacy and ethical restrictions.
